# Can we resist another person’s gaze?

**DOI:** 10.3389/fnbeh.2015.00258

**Published:** 2015-09-30

**Authors:** Barbara F. M. Marino, Giovanni Mirabella, Rossana Actis-Grosso, Emanuela Bricolo, Paola Ricciardelli

**Affiliations:** ^1^Department of Psychology, University of Milano-BicoccaMilan, Italy; ^2^Department of Physiology and Pharmacology “V. Erspamer”, La Sapienza UniversityRome, Italy; ^3^IRCSS NeuromedPozzilli, Italy; ^4^Milan Center for NeuroscienceMilan, Italy

**Keywords:** voluntary motor control, behavioral flexibility, saccadic eye movements, countermanding task, gaze-following behavior, social attention, saccadic inhibition

## Abstract

Adaptive adjustments of strategies are needed to optimize behavior in a dynamic and uncertain world. A key function in implementing flexible behavior and exerting self-control is represented by the ability to stop the execution of an action when it is no longer appropriate for the environmental requests. Importantly, stimuli in our environment are not equally relevant and some are more valuable than others. One example is the gaze of other people, which is known to convey important social information about their direction of attention and their emotional and mental states. Indeed, gaze direction has a significant impact on the execution of voluntary saccades of an observer since it is capable of inducing in the observer an automatic gaze-following behavior: a phenomenon named social or joint attention. Nevertheless, people can exert volitional inhibitory control on saccadic eye movements during their planning. Little is known about the interaction between gaze direction signals and volitional inhibition of saccades. To fill this gap, we administered a countermanding task to 15 healthy participants in which they were asked to observe the eye region of a face with the eyes shut appearing at central fixation. In one condition, participants were required to suppress a saccade, that was previously instructed by a gaze shift toward one of two peripheral targets, when the eyes were suddenly shut down (social condition, SC). In a second condition, participants were asked to inhibit a saccade, that was previously instructed by a change in color of one of the two same targets, when a change of color of a central picture occurred (non-social condition, N-SC). We found that inhibitory control was more impaired in the SC, suggesting that actions initiated and stopped by social cues conveyed by the eyes are more difficult to withhold. This is probably due to the social value intrinsically linked to these cues and the many uses we make of them.

## Introduction

Performing an action brings about a cost, thus animal brains have evolved extensive networks which first evaluate, and then select those behaviors that are more likely to enhance their biological fitness. This task might be extremely hard, as we live in an ever changing environment, where the value of a given action is not fixed, but depends upon the contextual situation. For instance, both looking at the eyes of a potential partner and avoiding looking at the eyes of a person we are ashamed of might represent highly rewarding actions. The evaluation process must be performed both during the initiation of an action as well as during its planning (for a review, see Mirabella, 2014). In fact, during the temporal gap between the time when an action has been chosen and the moment when the motor output is about to be generated, the environmental circumstances might have changed, requiring a radical modification of the planned motor strategy. There are two executive functions central to the implementation of flexible behaviors: (i) the ability to predict future outcomes of actions and (ii) the ability to cancel them when they are unlikely to accomplish valuable results. The evaluative process is greatly influenced by the stimuli that are encountered in the environment. In particular, emotional and social stimuli tend to be very salient, as they convey highly relevant information for our survival.

Among social stimuli the gaze of other individuals is a crucial one. Indeed, since birth people have an innate sensitivity to the gaze of others (e.g., Farroni et al., 2002, 2004). Gaze direction can have multiple meanings (e.g., signaling where another person is attending to), and serves both as an attentional cue and as a social signal. Perceiving the direction of somebody else’s gaze leads the observer to shift his/her attention in the same direction or towards the same object that the other person is looking at. This phenomenon is known as social or joint attention and it can be achieved both overtly, i.e., through eye movements (gaze-following behavior), and/or covertly, i.e., through an automatic and reflexive shift of spatial attention (gaze cueing effect; for a review, see Frischen et al., 2007). Its role is to allow us to understand other people’s intentions and actions and to create a perceptually shared ground of what we and others see, attend to and experience so as to enable social interaction (e.g., Moore and Dunham, 1995). All of this makes the gaze of others a special and powerful cue.

Indeed, joint attention is maintained in adulthood and to be overridden requires top-down control processes (e.g., see Ricciardelli et al., 2013). In fact, it has been shown that perception of gaze direction interferes with the execution of voluntary saccades (e.g., Ricciardelli et al., 2002, 2009, 2013; Nummenmaa and Hietanen, 2006). For instance, in one experiment observers were instructed to perform goal-directed saccades towards one of two peripheral stationary targets (Ricciardelli et al., 2002). A task irrelevant face was presented at fixation. The direction of the distracter’s gaze could match (congruent condition) or conflict (incongruent condition) with the direction of the instructed saccade. A substantial number of incorrect saccades matching the direction of the distracting gaze (gaze-following errors) were found. This was particularly the case when the distracting gaze shortly preceded the instruction to saccade and was reduced (or absent) when it followed it. The authors interpreted these findings as evidence that observed gaze, automatically and involuntary, evokes saccade preparation in the same direction to which the gaze points. These results were confirmed in a study by Kuhn and Kingstone (2009). In their Experiment 1, they also showed that non-predictive eye gaze decreased participants’ voluntary saccade latencies and increased accuracy when targets where congruent with gaze direction (see also Deaner and Platt, 2003; Mansfield et al., 2003). The automatic nature of the interference effect of gaze cues on volitional saccades was further confirmed in Experiment 2 in which they informed participants that gaze cues were counter predictive of the target’s true location. Even under these conditions, gaze cues could not be ignored and participants were still faster and more accurate when gaze direction was congruent with the target of the saccade. Therefore like non-social exogenous cues (such as a change in luminance of a target), gaze cues are relatively insensitive to participants’ intentions, i.e., they are resistant to top-down control. Recent studies, however, have shown that gaze-following behavior and gaze cueing are not completely reflexive, but depend in part on socio-cognitive variables, such as social status (Dalmaso et al., 2012), environmental context (Ricciardelli et al., 2013), familiarity (Deaner et al., 2007) as well as on physical features such as facial masculinity (e.g., Ohlsen et al., 2013) and face age (Ciardo et al., 2013, 2014). In other words, the gaze-shifts of some people are more valuable than others according to internally driven factors. Moreover, mental state attribution and beliefs about the minds of others can influence how we process, select relevant information and orient to it (e.g., Teufel et al., 2010; Wiese et al., 2012; Wykowska et al., 2014; Gobel et al., 2015; Richardson and Gobel, 2015).

It must be added, however, that the overall picture is far from being complete and clear. For instance, Koval et al. (2005) provided evidence that the information conveyed by gaze direction signals, when in conflict with task demand, can be efficiently suppressed so as to avoid unwanted gaze-following behavior. Specifically, to determine the effect of observed gaze direction on saccade preparation, they employed an anti-saccade task, which required the generation of a voluntary saccade in the direction opposite to stimulus location. In some trials, participants were instructed by a cue to make a saccade towards a peripheral target (pro-saccade), while in others to make a saccade away from it to its mirror location in the opposite visual field (anti-saccade). Two hundred milliseconds after cue onset, a task irrelevant face with averted gaze was presented at the center of the screen, followed by the onset of the peripheral target 500 ms later. Peripheral targets could be congruent or incongruent with the gaze cue. The authors found a facilitation of performance, (i.e., shorter reaction times, RTs), for the gaze congruent condition in the pro-saccade trials, but not for the gaze incongruent condition in the anti-saccade trials (for similar results, see Wolohan and Crawford, 2012). The authors interpreted this finding as evidence that participants prepared a saccade towards the observed gaze direction on pro-saccade trials, and away from the observed gaze direction on anti-saccade trials. Thus they suggested that a saccade triggered by the observed gaze can be under some strategic control. However, the anti-saccade task is in a sense ambiguous. In fact, it cannot be established whether, during anti-saccade trials, saccade preparation toward the direction of the observed gaze is subsequently modified, or is first suppressed and later a new saccade toward the opposite location is generated. A better understanding of the influence of gaze direction signals on voluntary saccade generation can instead come from the investigation of another type of top-down control, which is at the heart of voluntary movement control, i.e., volitional inhibition (for a review, see Mirabella, 2014). To the best of our knowledge, the influence of gaze direction on this executive function has never been investigated before. Volitional inhibition allows the suppression of planned actions when unexpected changes in the external environment or in our thoughts occur during the temporal gap starting from the instant when the initial decision whether to act is taken to the instant when the motor output is about to be generated. Both humans and non-human primates are capable of exerting volitional inhibition on saccades as shown by several studies exploiting the countermanding task (or stop signal task, e.g., Hanes and Schall, 1996; Hanes et al., 1998; Hanes and Carpenter, 1999; for a review, see Schall, 2013). The countermanding paradigm yields an estimate of the duration of the suppression process (stop-signal reaction time; SSRT), a phenomenon which is not directly measurable but which can be estimated exploiting the race model (for details, see Logan and Cowan, 1984). Thus, we thought of using this parameter to compare the inhibitory control of an oculomotor program in two different conditions, i.e., when the saccade was initiated by the gaze shift of another person and stopped by the eyes closure (social condition, SC), vs. when it was instructed by a change in color of a peripheral target, and stopped by the change of color of a centrally presented picture (non-social condition, N-SC). If eye-gaze cues are powerful trigger signals, since they convey crucial social information to the observer, then we can expect that the inhibition of saccades triggered by them will be more difficult than those instructed by non-social cues.

### The Choice of the Go-Stimuli

Attentional orienting can be obtained either reflexively (exogenous orientation) or volitionally (endogenous orientation, see Jonides, 1981). Traditionally, it was assumed that reflexive orienting could be achieved only in response to sudden stimulus changes, such as the abrupt onset of a peripheral cue. However, similar to reflexive cues, gaze direction, although centrally presented, triggers attentional orientation even when it is spatially non-predictive (e.g., Friesen and Kingstone, 1998). Thus, attentional orienting in response to gaze direction signals fulfills the main characteristics traditionally associated with reflexive attentional orienting, allowing us to compare peripheral and central go-signals. Nevertheless, it must be stressed that in some contexts attentional orienting in response to centrally presented gaze direction signals may be under some voluntary control in comparison to cues presented in the periphery (e.g., Frischen et al., 2007). All in all, gaze signals seem to be able to orient attention both exogenously and endogenously according to the task at play.

Recent findings have shown that, like gaze direction, even centrally presented cues like arrows might induce exogenous attentional orientation (for a review, see Frischen et al., 2007). Nevertheless, there are differences in the effects produced by central arrows and peripheral cues. For instance, the directional information conveyed by arrows can be suppressed if that information conflicts with task demands, indicating that orienting to central cues is less automatic than orienting to peripheral cues (Friesen et al., 2004).

At least in principle, the central eye gaze cue is likely to be a more powerful and automatic attentional cue than central arrows, because it is conceivable that we evolved a neural architecture specialized for eye processing (Baron-Cohen, 1995). However, it is also plausible that in laboratories, but not in the real world, biologically irrelevant stimuli, such as central arrows, might act as go-signal similar to gaze direction. This possibility could be addressed in future studies.

In any case, given the many contradictory studies on the real automaticity of attention orienting in response to central arrows (e.g., Ricciardelli et al., 2002; Langdon and Smith, 2005; Kuhn and Kingstone, 2009; Gregory and Hodgson, 2012), we chose to employ a typical exogenous cue (a change in color of a peripheral cue) as a control condition for our social cue, as most studies assert that attentional orienting is highly similar in these two situations.

## Materials and Methods

### Participants

Fifteen undergraduate students (12 female, 3 male, mean age = 21.8 years, SD = 2.0) from the University of Milano-Bicocca received course credits for their participation in the study. All had normal or corrected-to-normal vision, had no history of neurological diseases, and were unaware of the study’s purpose.

All participants gave written informed consent before testing. The study was conducted in accordance with the ethical standards laid down in the 1964 Declaration of Helsinki and fulfilled the ethical standard procedure recommended by the Italian Association of Psychology (AIP). All the experimental protocols were also approved by the Ethics Commission of Milano-Bicocca University.

### Apparatus and Materials

The experiment was carried out in a sound-attenuated room, dimly illuminated. Participants sat approximately 116 cm away from a 27-inch LCD monitor (acer^®^ HN274H; Resolution: 1920 × 1080 pixels; Refresh rate: 120 Hz) with their head placed on a chinrest in order to maintain a stable eye-to-screen distance. The monitor was interfaced with an AMD Athlon^™^ Dual Core 2.00 GHz personal computer equipped with a NVIDIA^®^ GeForce^®^ GTX 560 Video Board. The experimental apparatus also comprised an infrared remote/head-free eye-tracking system (EyeLink 1000^®^, SR Research Ltd.) with a recording spatial resolution of 0.01 degree of visual angle (hereafter degree) and a sampling rate of 1000 Hz.

Three grayscale photos (4.31° × 1.48°) of the eye region of one of the authors (P.R.), bearing a neutral expression, were used as gaze stimuli. The photos depicted, respectively, a closed gaze (with eyelids closed over both eyes), a leftwards gaze (with visible irises and pupils in the left-most position of the eye sockets), and rightwards gaze (with irises and pupils in the right-most position of the eye sockets).

### Procedure

The participants were individually tested in two experimental sessions, one for each experimental condition (SC and N-SC, see below for a detailed description). The two sessions were administered on different days, at least 72 h apart. Their presentation order was counterbalanced across participants, thus half of the participants performed the SC first, and *vice versa* for the other half.

In each experimental session, which lasted about 70 min, participants were asked to perform two tasks: (a) a go-only saccade task, and (b) a saccade countermanding task (e.g., Hanes and Schall, 1996), while the movements of their dominant eye were recorded. The two tasks were presented in separate blocks and the order of presentation was counterbalanced across the participants. Resting periods were allowed between blocks whenever requested.

**Figure 1:**
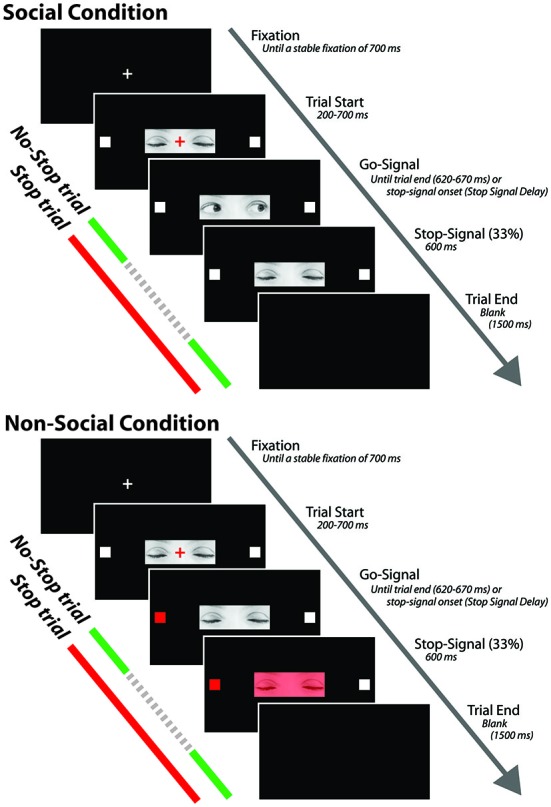
**Schematic representation of the experimental procedure for both social and non-social conditions.** The stop signal is presented only in the stop trials and not during the no-stop trials. The dashed portion of the arrow indicates that stop signals are not shown during no-stop trials (see text for further details).

Each trial started with the presentation of a white fixation cross (0.35° × 0.35°) centrally displayed on a black background (Figure 1). After a stable fixation of 700 ms, the color of the cross turned red and the photo depicting the closed gaze was centrally presented on the screen so that the cross laid on the between-eyes point of the closed gaze. The gaze was flanked by two white target squares (0.7° × 0.7°), one to the left and the other to the right of the horizontally aligned fixation cross (eccentricity: 7.91°). After a variable delay of 200–700 ms (in order to avoid response habituation), the red fixation cross was switched off and the go-signal was delivered. If participants made a saccade before the go-signal onset the trial was aborted and recycled at the end of the trial block. In the go-only trials of the SC, the go-signal consisted of a dynamic gaze, shifting randomly towards the left or the right target: the gaze shift was created by replacing the photo of the closed gaze with the photo of either leftwards or rightwards gaze. By contrast, in the go-only trials of the N-SC, the go-signal consisted of a change in color from white to red, of either the left or the right target. The participants were required to make a fast and accurate saccade towards the looked-at target (in the SC) or towards the target that changed color (in the N-SC), and to maintain fixation on this target as long as it was visible (i.e., for about 620–670 ms).

In both the SC and the N-SC, the countermanding task (Figure 1) consisted of a random mix of 67% no-stop trials and 33% stop trials. No-stop trials were identical to go-only trials. Stop trials differed from the no-stop trials because at a variable delay (stop-signal delay, SSD) after the presentation of the go-signal a stop signal appeared, indicating to participants that they should inhibit their saccade. In the SC, the stop-signal consisted of the closure of the eyes that was created by replacing the photo of the adverted gaze with the photo of the closed gaze. In the N-SC, the stop-signal consisted of a change in color of the closed gaze due to the superimposition a semi-transparent red filter on the original photo. Participants were instructed to inhibit the saccade when the stop-signal was presented and to maintain a steady central fixation until the end of the trial (i.e., until 600 ms had passed). Trials in which participants successfully withheld the movement were defined as stop-success trials and those in which they moved were defined as stop-failure trials. Error trials were not repeated. A blank screen for 1500 ms was presented during the inter-trial interval.

The length of the SSDs was controlled by using a QUEST adaptive stair-case procedure (Watson and Pelli, 1983) with a 50% performance criterion. The β, δ and γ parameters of the Weibull psychometric function were set to 3.5, 0.01, and 0.5, respectively, as pilot studies indicated that these values were appropriate for obtaining the desired amount of inhibition in stop trials. Before starting the countermanding task, participants were informed that in some stop trials they would not be able to cancel the movement and that they should not be concerned by this. In addition, it was emphasized that they give the same importance to stop and no-stop trials, and to perform fast saccades towards the peripheral target in no-stop trials.

In each experimental session, the go-only saccade task consisted of one block of 100 trials, plus 16 practice trials. The countermanding task consisted of a block of 360 trials (240 no-stop trials and 120 stop trials), plus an initial block of 18 practice trials. To sum up, the whole experiment consisted of a total of 988 trials. Throughout the countermanding and go-only tasks, participants could take a break, if needed, after every 36 and 25 trials, respectively.

Timing, stimulus presentation, SSD computation and response collection were controlled by using the Psychophysics Toolbox Version 3 (PTB-3; Brainard, 1997; Pelli, 1997) within MatLab (R2010a) programming environment. Just before the beginning of each task and after each break, the eye-tracking system was calibrated using the EyeLink1000 five-points standard procedure.

### Data Analysis

Practice trials were discarded from the analysis. Eye movements data, i.e., eye positions in calibrated screen pixel coordinates, were analyzed off-line. The participants’ oculomotor behavior performed in each trial was parsed using the EyeLink Data Viewer application, which allows identification of saccades and fixations by means of a combined eye velocity/acceleration criterion. In particular, saccade onsets and offsets were detected by a velocity threshold of 30°/s and an acceleration threshold of 8000°/s^2^. This criterion allowed reliable identification of all saccades larger than 0.6°. The EyeLink Data Viewer was also used to compute raw saccadic latencies, amplitudes, directions, peak velocities, and durations. For each trial, the first horizontal eye movement that followed the go-signal presentation and exceeded 1.76° of amplitude was considered a saccade. The remaining trials were then screened for errors. We classified as errors and discarded from further analyses: (a) no-stop/go-only trials in which eye responses were directed away from the instructed targets (i.e., antisaccades, 0.3% of total go trials); (b) no-stop/go-only trials with missing responses, i.e., when participants did not move the eyes (2.5% of total go trials); and (c) stop trials with antisaccades (0.6% of total stop trials). Response selection and error screening procedures were implemented by an algorithm within R environment for statistical computing (version 3.1.1; R Development Core Team, 2014). The same algorithm also computed for each subject and each experimental condition the median value of saccadic latency and duration as well as the saccadic average amplitude, and peak velocity, allowing a complete kinematics analysis of eye responses.

To quantify the ability of participants to inhibit saccade production, we estimated the SSRT for each subject and for each experimental condition exploiting the so-called “integration model” (e.g., see Federico and Mirabella, 2014). This method is based on the independence assumption of the race model (Logan and Cowan, 1984), which implies that the distribution of RTs on stop trials (whether a response is canceled or not) is the same as the distribution of RTs of no-stop trials. Thus the SSRT is obtained by subtracting the starting time of the stop process from its finishing time (Logan and Cowan, 1984; Band et al., 2003). The starting time of the stop process is given by the mean SSD, which was computed using the mid-run estimates method (see Levitt, 1971). This method has been shown to estimate reliably the mean value of a variable manipulated with a staircase procedure. The finishing time of the stop process was calculated by integrating the no-stop trials RT distribution from the onset of the go-signal until the integral equals the corresponding observed proportion of stop-failure trials.

Finally, to further characterize mechanisms of saccade initiation and control in the two countermanding conditions, we also considered the distribution of saccadic latencies recorded in stop-failure trials.

### Statistics

Saccadic latency, amplitude, duration, and peak velocity were separately submitted to a two-way repeated measures ANOVA with trial type (go-only trials, no-stop trials, stop-failure trials) and condition (SC, N-SC) as factors. Mauchley’s test evaluated the sphericity assumption and where appropriate, correction of the degrees of freedom was made according to the Greenhouse–Geisser procedure. When needed, *post hoc* tests (pairwise comparisons) with Bonferroni correction were employed. Estimates of SSRT were submitted to a paired *t*-test with Condition (SC, N-SC) as the within-subject variable. Additionally, the Kolmogorov–Smirnov test was exploited for contrasting cumulative distributions of saccadic RTs obtained in no-stop, stop-failure, and go-only trials. In order to control for the sample size, we computed the eta-squared (η^2^) for each ANOVA and the Cohen’s *d* for *t*-tests (see Lakens, 2013). Both coefficients estimate the so-called “effect size”, i.e., a measure describing the degree of relationship between dependent and independent variables independently of the sample size. Values of η^2^/Cohen’s *d* higher than 0.14/0.8 indicate strong effect sizes, namely that the *F*-values/*t*-values obtained are unlikely to depend on the sample size. Values of η^2^/Cohen’s *d* around 0.06/0.5 indicate medium effect size, and values smaller than 0.01/0.2 indicate small effect sizes (Cohen, 1988; Lakens, 2013).

## Results

### Effects of Social and Non-Social Signals on the SSRT

The main aim of this study was to assess the effect of triggering a saccade exploiting either a social cue or a non-social cue as go-and stop-signals on inhibitory control. To this end, we estimated the length of the inhibitory process in the two conditions. First of all, the estimation of SSRT was reliable. In fact, the staircase algorithm kept the average proportion of stop-failure trials [*p*(failure)] at the desired value (i.e., ~0.5, see Table 1; see also Band et al., 2003) in both conditions (paired *t*-test *t*_(14)_ = 1.1, *p* = 0.31). In addition, we checked whether task performance met the assumptions of the independent race model, i.e., whether the saccadic RTs of stop-failure trials should be shorter than those of no-stop trials (Logan et al., 1984; Logan, 1994; Boucher et al., 2007). Therefore, for each participant and for each task condition we computed how many times the distributions of the saccadic RTs of stop-failure trials were significantly different from those of no-stop trials. We found that 12 out of 15 (or 80%, *χ*^2^ = 5.4, *p* < 0.05), and 13 out of 15 (or 86.6%, *χ*^2^ = 8.1, *p* < 0.01) participants in the SC and in the N-SC, respectively, fulfilled the model assumption (Kolmogorov–Smirnov test, all *p* < 0.05). Overall, these results indicate that our data gave a good estimate of the SSRT.

**Table 1:** **Summary of behavioral results for social and non-social conditions**.

	Social condition	Non-social condition
Mean SSD	205.3 (22.3)	226.3 (19.4)
P(failure)	0.54 (0.02)	0.53 (0.01)
SSRT	144.3 (11.7)	112.7 (8.4)
RT no-stop trials	329.2 (9.2)	332.7 (13.7)
RT stop-failure trials	292.7 (7.2)	289.8 (12.5)
RT go-only trials	250.9 (10.4)	226.9 (5.9)

All mean (SE) values, except for P(failure), are expressed in milliseconds. Abbreviations. RT, saccadic reaction time; SSRT, stop-signal reaction times; SSD, stop-signal delay (see text for further details).

**Figure 2:**
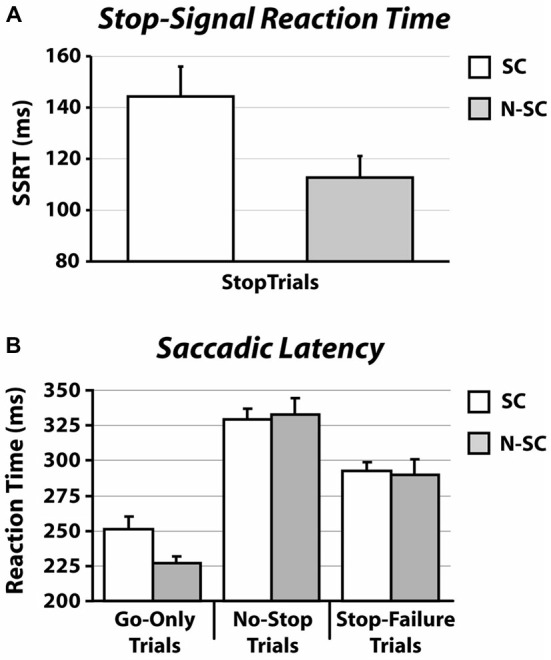
**SSRT (A) and RT (B) in for social and non-social conditions.** Panel **(A)** shows the average SSRT. Panel **(B)** illustrates the average RT (SE) of go-only trials, no-stop trials and stop-failure trials. Error bars represent the standard error.

Importantly, as shown in Figure 2A and Table 1, the SSRT in the SC condition was longer than that measured in the N-SC condition (paired *t*-test, *t*_(14)_ = 3.1, *p* < 0.01, Cohen’s *d* = 0.8), indicating that the nature of the go- and/or the stop-signal significantly impacts the ability to cancel pre-planned saccades.

### Effects of Social and Non-Social Signals on the Saccadic RT

**Figure 3:**
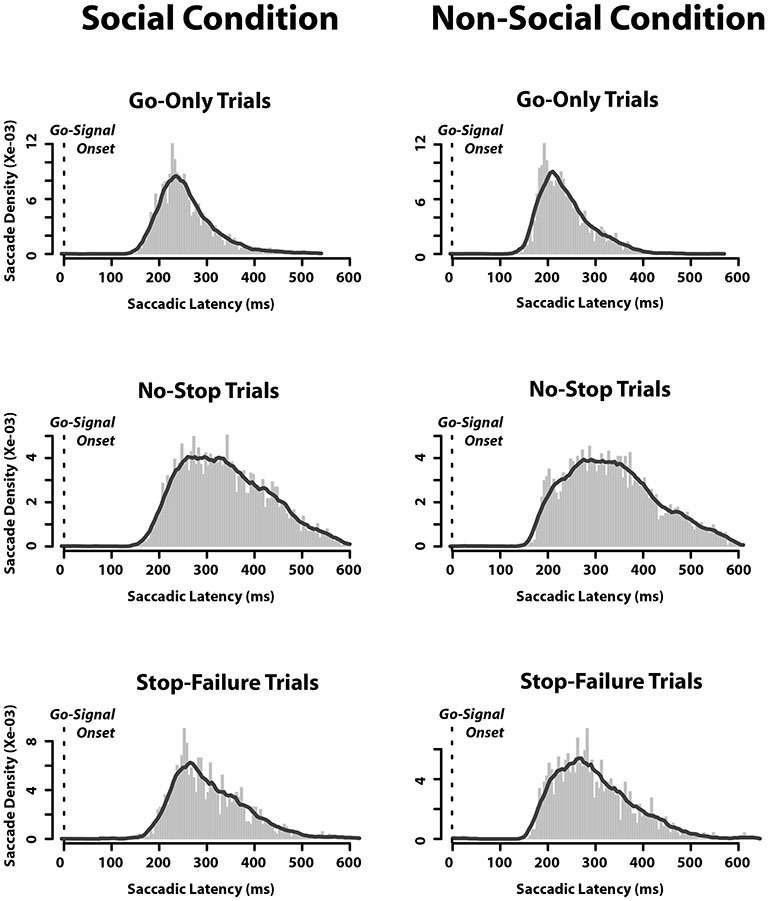
**Saccadic latency distributions in each type of trials in which an eye movement was executed in both social and non-social conditions.** Distributions are aligned as a function of time elapsed since the appearance of the go-signal (bin size = 5 ms).

As shown in Figures 2B, 3 and Table 1, the nature of the go-signal did not affect the RTs of saccades. A 2-way repeated-measures ANOVA [factors: trial type (3 levels: go-only, no-stop, and stop-failure trials) and condition (2 levels: SC and N-SC)] revealed only a main effect of trial type (*F*_(2, 28)_ = 95.5, *p* < 0.0001, η^2^ = 0.5). The *post hoc* tests (pairwise comparisons with Bonferroni correction) indicated that, overall, saccadic RTs of go-only trials were significantly shorter than those of both no-stop trials (*p* < 0.0001) and stop-failure trials (*p* < 0.0001). This result is in line with the well-known phenomenon of the delay strategy (e.g., Logan, 1981; Mirabella et al., 2006, 2008, 2013; Verbruggen and Logan, 2009). This is a form of proactive control (i.e., a control over response execution in anticipation of known task demands) that participants adopt when they are aware of the presence of the stop-signal, in order to maximize the number of correct responses to the stop trials. In addition, the RTs of stop-failure trials was shorter than that of no-stop trials (pairwise comparisons with Bonferroni correction, *p* < 0.0001), indicating that even at population level the race model assumptions were satisfied.

At least in principle, the effects of gaze cue on RTs might be more pronounced at the beginning of the session, because of the coupling between the novelty and the saliency of this stimulus. Thus, to check whether the gaze cue undergoes habituation over the course of the session, we considered the RTs of no-stop trials and go-only trials. For each participant, we averaged the RTs across groups of ten trials. As participants completed at least 220 no-stop trials and 90 go-only trials, for each condition we had 22 and 9 points respectively (see Table 2). To compare RTs across the SC and N-SC, we ran two separate 2-way repeated-measures ANOVA [factors: blocks (22 levels) and condition (2 levels: SC and N-SC)] one for blocks of no-stop trials and the other for blocks of go-only trials. None of the ANOVAs revealed any significant effects, indicating that both the social and the non-social cue did not show habituation, thus the effects found are long lasting and they do not die away after the first blocks of trials. Possibly, this is due to the fact that no-stop trials were randomly intermixed with stop trials, and this variability prevented any form of habituation.

**Table 2:** **Average reaction times of subsequent blocks of no-stop/go-only trials for social (SC) and non-social conditions (N-SC)**.

	No-stop trials SC	No-stop trials N-SC	Go-only trials SC	Go-only trials N-SC
Block 1	329.51 (28.4)	298.45 (17.1)	276.01 (13.7)	248.81 (8.2)
Block 2	349.13 (31.0)	330.15 (20.3)	258.79 (14.3)	247.31 (8.5)
Block 3	338.73 (22.5)	317.71 (24.9)	255.79 (11.5)	235.23 (8.3)
Block 4	351.17 (23.2)	342.93 (22.0)	265.23 (11.6)	241.99 (8.2)
Block 5	329.53 (22.3)	328.89 (22.6)	256.25 (10.5)	246.46 (9.3)
Block 6	338.73 (20.5)	329.67 (22.7)	259.16 (9.9)	239.69 (7.0)
Block 7	343.07 (17.4)	338.06 (25.9)	257.23 (11.7)	231.72 (6.1)
Block 8	356.86 (23.6)	345.15 (23.7)	241.68 (7.4)	226.55 (7.1)
Block 9	359.90 (23.3)	353.27 (21.9)	251.49 (8.1)	227.96 (8.7)
Block 10	345.38 (20.9)	327.81 (19.2)		
Block 11	329.16 (17.3)	332.73 (19.6)		
Block 12	338.28 (25.3)	340.89 (27.4)		
Block 13	333.01 (15.5)	339.02 (27.9)		
Block 14	344.87 (16.2)	331.76 (28.4)		
Block 15	327.85 (22.1)	333.45 (24.9)		
Block 16	339.79 (16.3)	331.03 (22.5)		
Block 17	324.05 (23.4)	322.71 (23.8)		
Block 18	316.63 (26.9)	348.14 (31.3)		
Block 19	322.08 (22.4)	351.41 (29.8)		
Block 20	338.99 (18.8)	341.62 (20.7)		
Block 21	341.75 (19.3)	346.82 (13.5)		
Block 22	328.38 (12.5)	341.64 (14.4)		

All mean (SE) values are expressed in milliseconds.

Finally, we also assessed the saccadic latency distributions. We found that when they were aligned to the appearance of the go-signal, there was no difference between the SC and the N-SC (Figure 3). As expected, the distributions of saccadic latency of no-stop trials were wider than those of go-only trials, while those of stop-failure trials were half a way between the two.

**Figure 4:**
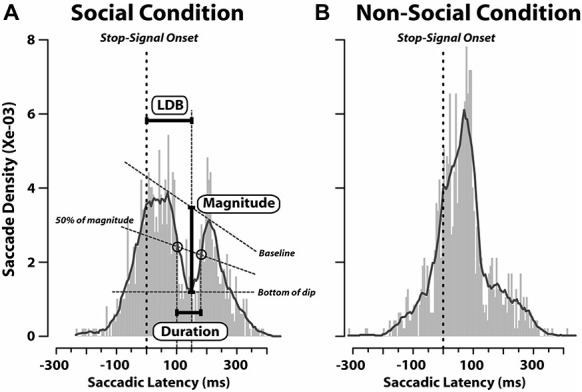
**Saccadic latency distributions in stop-failure trials for social (A) and non-social conditions (B).** Distributions are aligned as a function of time elapsed since the onset of the stop-signal (vertical dashed red lines; bin size = 5 ms). In each plot, a black line represents the probability density function which was computed by applying a smoothing kernel (bandwidth = 3) to the saccadic latency distribution. The baseline is a straight line connecting the two peaks. The bottom of the dip indicates the minimum density inside the dip. The magnitude of decrease in saccade latency is given by the difference between the density at the baseline value and the density at the bottom of the dip (Latency to Dip Bottom, LDB). The line named “50% of magnitude” represents the latency from the stop-signal onset at which the decrease achieved 50% of its magnitude along each peak.

Nevertheless, since it has been found that when a visual task-irrelevant stimulus (such as a flash or flicker) appears shortly after the target presentation, it causes a decrease in the probability of saccadic initiation (e.g., Reingold and Stampe, 2000, 2002) we computed also the latency distribution of saccades aligned to the appearance of the stop-signal in stop-failure trials (Figure 4). We performed this analysis on stop-failure trials, because these are the only ones in which, despite the stop-signal having been presented, participants erroneously produced a saccade. In this context, the stop-signal might act as a distracting stimulus, which might conflict with the target towards which the saccade is about to be initiated. We aimed to check whether or not this phenomenon occurred both in the SC and N-SC. As can be noted from Figure 4, this was the case only in the SC, suggesting that only the closure of the eyes is treated by the observer’s oculomotor system as a relevant cue. In this condition, in fact, latency distribution of responses exhibited a dip, with a magnitude of 0.002, where magnitude was defined as the amount of decrease in saccadic density measured as the difference between the density at the baseline value and the density at the bottom of the dip (see Figure 4). The dip started around 100 ms after the stop-signal onset, reached its maximum at 150 ms, and lasted about 80 ms (corresponding to the time interval during which the decrease of saccadic density was equal to or greater than 50% of its magnitude). This was the only analysis which revealed differences in saccadic RTs between the SC and the N-SC.

### Effects of Social and Non-Social Signals on Kinematics of Saccades

**Figure 5:**
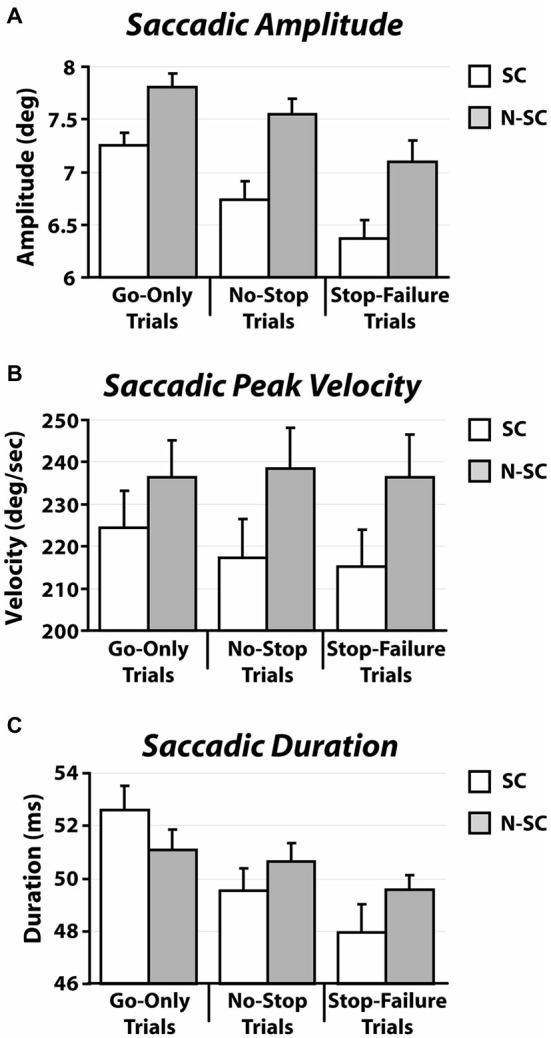
**Kinematics [amplitude (A), peak velocity (B), and duration (C)] of saccadic responses for both social and non-social conditions recorded in go-only trials, no-stop trials and stop-failure trials.** Error bars represent the standard error.

In contrast to what we found for saccadic RTs, the nature of the go-signal affected the kinematics of saccades (amplitude, peak velocity and saccadic duration; Figure 5 and Table 3).

**Table 3:** **Summary of kinematic parameters of saccades for social and non-social conditions**.

	Amplitude (degree)		Duration (ms)		Peak velocity (degree/s)	
	SC	N-SC	SC	N-SC	SC	N-SC
Go-only trials	7.2(0.1)	7.8(0.1)	52.6(1.1)	51.1(0.9)	224.4(10.2)	236.4(10.7)
No-stop trials	6.7(0.2)	7.5(0.2)	49.2(0.9)	50.6(0.8)	217.2(10.7)	238.4(11.4)
Stop-failure trials	6.3(0.2)	7.1(0.2)	47.9(1.2)	49.5(0.7)	215.1(10.2)	236.4(11.7)

All values represent mean (SE). Amplitude is expressed in degrees of visual angle, Duration is expressed in milliseconds (ms), and Peak velocity is expressed in degrees per second.

First of all, a 2-way repeated-measures ANOVA on the amplitude of saccades with trial type (3 levels: go-only, no-stop, and stop-failure trials) and condition (2 levels: SC and N-SC) was performed. It revealed both a significant main effect of trial type (*F*_(2, 28)_ = 33.6, *p* < 0.0001, η^2^ = 0.256) and condition (*F*_(1, 14)_ = 15.3, *p* < 0.0001, η^2^ = 0.246). Overall participants undershot the target of an amount that varied depending on both condition and trial type. The main effect of the factor condition was due to the fact that overall saccadic undershooting was larger in SC than in N-SC. In addition, the main effect of trial type indicated that undershooting differed across the type of trials. In fact, the *post hoc* test (pairwise comparisons with Bonferroni correction, all *p*s < 0.001) showed that the undershooting progressively increased from go-only trials to no-stop trials, and to stop-failure trials.

Secondly, a 2-way repeated-measures ANOVA with the same factors as before was performed on saccadic peak velocity. It revealed only a main effect of the factor condition (*F*_(1, 14)_ = 10.1, *p* < 0.01, η^2^ = 0.415), as saccades during N-SC were faster.

Finally, the same factorial design was used for a repeated measure ANOVA on saccadic duration. We found both a main effect of factor trial type (*F*_(2, 28)_ = 18.7, *p* < 0.0001, η^2^ = 0.114) and of the interaction between trial type and condition (*F*_(2, 28)_ = 4.8, *p* < 0.05, η^2^ = 0.033). The main effect of trial type was due to the fact that the duration was longest in go-only trials with respect to that in all other types of trials (pairwise comparisons with Bonferroni correction, all *p*s < 0.005). In addition, the duration of saccades in no-stop trials was also longer than in stop-failure trials (*p* < 0.05). The interaction indicated that saccades took longer in go-only trials of the SC than those executed during no-stop trials of the SC (*p* < 0.01), and longer than those executed in stop-failure trials of both the SC and the N-SC (all *p*s < 0.01). Furthermore saccades of go-only trials in the N-SC took longer than those executed in the stop-failure trials of the SC (*p* < 0.005) and saccades in no-stop trials of SC took longer than those of stop-failure trials of the same condition (*p* < 0.05).

### Effects of Perceptual Load of Go Cues on RT and Inhibitory Control

As the social cue appears to be a more complex stimulus than the exogenous cue, it might be argued that the visual analysis of the former stimulus might bear a larger perceptual load. In other words, detecting the direction of gaze might engage additional processing compared to detecting a simple color change that may have nothing to do with social information. If this is the case, it might be suggested that the difference in the SSRT that we found could be partially or completely due to this additional visual processing time. In our view, this hypothesis is unlikely because an increased perceptual load should have been reflected in a significant lengthening of RTs of no-stop trials in the SC compared to the N-SC, but this was not the case. However, in order to directly address this issue, we administered an additional experiment on 16 participants (see Supplementary Material). The experiment consisted of three versions of a go/no go task in which participants upon the onset of the go signal were required to determine: (1) the direction of the shifting gaze (in the SC); (2) the location (left or right) of the target that changed color (in the peripheral N-SC); and (3) the location (left or right) of the eye with the bigger pupil (for half of the participants) or the smaller one (for the other half of participants), by pressing spatially corresponding keys (i.e., left or right) with their index fingers on the computer keyboard. In the first two tasks, we employed the same go-signals used in the main experiment (i.e., the gaze cue and the peripheral cue). By contrast, in the third version, the go-signal consisted of the opening of the eyes which was done by replacing the photo of a closed gaze with the photo of a direct gaze displaying the left or right pupil bigger than the other pupil. Thus, this was a non-social stimulus, presented centrally, which aimed to bear a perceptual load close to that of the go-signal employed in the SC. Crucially, we found that the RTs of correct go-trials were longer when this last type of go-signal was employed compared to when the gaze shift or the lighting up of peripheral targets were used (see Supplementary Material for further details). Again, as already shown for the main experiment, the detection of the direction of the gaze and the change in peripheral target color did not yield different average RTs, indicating that they require a similar amount of perceptual processing. All in all, these results suggest that the lengthening of the SSRT found in the main experiment for SC relative to N-SC is unlikely to be ascribed to a different perceptual load of the two go signals.

## Discussion

The main goal of the present study was to investigate whether it was more difficult to cancel a voluntary saccade initiated and stopped by social signals conveyed by the eyes, than one initiated and stopped by non-social exogenous cues. For this purpose, we used a saccadic countermanding task in which we compared the ability of participants to suppress a previously instructed saccade toward a peripheral target when it was triggered by the gaze shift of another person and stopped by the eyes closure, vs. when it was triggered by a change in color of a peripheral target, and stopped by the change of color of a central picture. We found that the nature of the signal used to initiate the saccade influences the time needed to cancel it. However, it did not affect the latency of saccades execution. Nevertheless, the nature of the go signal also exerted significant effects on saccadic kinematics.

### Effects of Gaze Direction Signals on Volitional Inhibition

In stop trials, suppressing saccades instructed by eye-gaze cues took longer, (see Figure 1), than suppressing color-instructed ones, suggesting that the inhibition of a saccade instructed by a social cue was more difficult and required more effort than suppressing a saccade instructed by a non-social cue. There are several potential explanations for this novel finding.

First, the mechanism underling this effect could be related to the way in which the two different go-signals trigger attentional orienting toward target locations. It is very well known that the sudden onset of a peripheral stimulus automatically orients attention to the location signaled by the spatial cue (Posner, 1980). Similarly, it has been shown that also gaze cues can produce an automatic orienting of attention in the direction indicated by the gaze (e.g., Friesen and Kingstone, 1998; Driver et al., 1999). Even though the two attentional orienting types present several differences, in particular the orienting in response to gaze direction emerges more slowly and lasts longer (Driver et al., 1999; Frischen et al., 2007), the fact that the nature of the go signal did not affect the saccadic latency makes such an account unlikely.

Secondly, it might be hypothesized that the difference in the SSRT may be related to the longer processing time required for the visual analysis of social cue compared to that needed to detect a simple color change. However, our control experiment (see Supplementary Material) revealed that our finding cannot be ascribed to a larger perceptual load of the social cue.

A third possibility is that the lengthening of SSRT in the SC is due to a difficulty in re-orienting attention back to the central stop-signal location once attention had been spatially oriented away by the averted gaze toward the peripheral target. Because gaze direction signals potential relevant information and by automatically orienting the observer’s attention, prioritizes the processing of the looked-at objects, it might also hold attention on the object location preventing it from going back to central location (a kind of inhibition of return). If this were the case, it might increase the time needed to detect the stop-signal, thus lengthening the SSRT. Although possible, this explanation does not fit with the fact that inhibition of return is usually not found when the gaze cue is presented along with the target (Friesen and Kingstone, 2003; for a discussion, see Frischen et al., 2007). Future research is needed to test this explanation exploiting, for example, peripheral stop-signal cues.

A fourth explanation could lie on the fact that only gaze direction conveys biological and social information (Baron-Cohen, 1995). Possibly, the resistance to cancel a movement may rest on the socially significant outcomes derived from looking at, and orienting attention in the same direction of the seen gaze. In other words, in the present study gaze direction, unlike the non-social signal, is associated with both the go-signal instruction, as well as to its intrinsic social value. This latter association, established through the experience of living in a social world, might make the cancelation of a saccade instructed by the gaze in the SC more difficult. Future research can test this explanation by changing the social value of gaze direction, for instance, by adding emotional expressions in order to see if the time needed to cancel a saccade instructed by an emotional gaze varies. Alternatively, the value of the peripheral non-social signal can be increased by adding a reward to it. In short, our data suggest that the difference in SSRT does not depend on whether the go-signal is a central or a peripheral cue, but rather on its intrinsic value. In fact, it has been shown that in an anti-saccade task where participants have to override the social value of gaze direction by programming a saccade in the direction opposed to the adverted gaze since the start of the trial, low-level peripheral cues and central gaze cues produce the same results (Fischer and Weber, 1996; Wolohan and Crawford, 2012).

Finally, the lengthening of the SSRT might also be ascribed to the nature of the stop-signal (which was social only in the SC) or to a combination of the effects elicited by the go- and the stop-signals. However, even if this is the case, the effects found in the present study are still likely to be due to the different feature (social vs. non-social) of the go and/or stop-signals. Future studies should disentangle their roles.

### Effects of Gaze Direction Signals on Saccade Generation

In the countermanding task when a saccade was executed, as in no-stop and stop-failure trials, no differences between SC and N-SC in terms of saccadic latencies emerged, suggesting that gaze and peripheral cues are equally effective in orienting attention to target location. Interestingly, the effect on RTs of SC with respect to N-SC was observed in the distribution of saccadic latency in stop-failure trials when aligned on the onset of the stop-signal. Just in the SC, the stop-signal provoked a decrease in the probability of producing a saccade in a time window of about 80 ms. Bearing in mind that in both conditions the stop-signal consisted of a visual stimulus centrally presented, this result indicates that only the closure of the eyes could interfere with the execution of erroneous saccades, while this was not the case for the change in color of the closed gaze (non-social stop-signal). These findings suggest that only sufficiently salient stimuli (either visual or social or both, and gaze direction is definitely one of them) can affect the likelihood of saccade production. Note that the closing of the eyes is a socially relevant signal since it can be interpreted as shyness, but also as rudeness or disengagement. Reingold and Stampe (2002) found somewhat similar results as they showed that flashing a visual stimulus (i.e., the sudden change in the color of two thirds of the monitor) shortly after target presentation, caused a decrease in the probability of producing a saccade. Evidently, in their setting the stimulus was sufficiently salient. By contrast, in our study, only the change occurring to the gaze cue (i.e., the eyes closing) was relevant enough to affect the probability of saccade generation. This underlies once again the higher saliency in terms of the social relevance of the gaze signal. In this respect it must be stressed that the different perceptual saliencies of the two stop-signals cannot account for the different inhibitory performance, because the staircase algorithm controls for it.

The difference found in RTs concerning the go-only and no-stop trials, consisting of faster RTs for go-only trials (see Figure 2), is a result in line with the countermanding manipulation which supports the reliability of our findings and the validity of our task. While performing the countermanding task, in fact, it is very plausible that participants constantly monitored for the appearance of the stop-signal that, in our study, appeared at the center of the screen in both conditions (see Figure 1). This “awaiting mode” may account for the lengthening of the RTs of no-stop trials with respect to go-only trials, the so-called delay strategy (e.g., see Logan, 1981; Mirabella et al., 2006, 2008, 2013; Verbruggen and Logan, 2009).

### Effect of Gaze Direction Signals on Saccadic Kinematics

Clear effects of the nature of the go-signal are evident in the saccadic kinematic results. In particular, gaze-instructed saccades had a lower velocity peak and systematically undershot the peripheral target indicating that the difference between the saccades generated by gaze direction and those generated by a non-social exogenous signal were not only quantitatively, but also qualitatively different. Specifically, a tendency to undershoot targets has been often reported in studies that used paradigms involving the presentation of a target along with a simultaneous distracting non-target (e.g., Ottes et al., 1985) or an irrelevant stimulus background (He and Kowler, 1989). In these cases, saccades land on a point somewhere between the target and the other stimulus. Similarly, saccadic undershooting in our study is plausibly due to the simultaneous presence of a central stimulus and of an eccentric target.

However the amplitude of saccades was modulated both across conditions and type of trials. The difference between SC and N-SC may result from the stronger tendency of participants to inspect open eyes rather than closed eyes. This hypothesis agrees with the fact that in the SC the RTs of go-only trials were longer than in the N-SC (see Figure 2). Possibly this effect is washed out in the no-stop trials and in the stop-failure trials, because of the large increase of RTs. The same argument, i.e., the tendency of exploring in depth the open eye gaze stimulus, might also explain the lower peak velocity of saccadic eye movements in the SC than in the N-SC. Further studies are needed to clarify this issue. In addition, the progressive decrease of saccadic amplitude from go-only trials, to no-stop trials and stop-failure trials may be the result of a proactive strategy that participants unconsciously adopt in order to monitor for the appearance of the stop-signal.

Finally, the duration of saccades are obvious consequences of a combination of their amplitudes and their velocity peaks. For instance, saccades executed during the go-only trials had the largest amplitude and the same velocity peak of the other two types of trials, and as a consequence had the longest duration. More relevantly, this analysis revealed, for the first time, that the so-called *context effect*, first shown for arm reaching movements (Mirabella et al., 2008, 2013), occurs also for saccades, at least to some extent. Mirabella et al. (2008) compared RTs and movement times (MTs) for reaching movements executed either during go-only trials and no-stop trials. As expected, the awareness of the presence of the stop-signal induced a lengthening of RTs for no-stop trials compared to those for go-only trials (e.g., see Logan, 1981; Mirabella et al., 2006, 2008, 2013; Verbruggen and Logan, 2009). In addition, Mirabella et al. (2008) showed a shortening of MTs in no-stop trials compared to those recorded in go-only trials. This phenomenon was interpreted as an optimization of motor strategy related to contextual information, because shorter RTs are compensated by longer MTs and* vice versa*. In contrast to saccades, arm movements are not ballistic movements (e.g., De Jong et al., 1990; Scangos and Stuphorn, 2010) and thus they can be stopped at any point along their path. Thus, the length of MTs could reflect the different need for on-line planning during go-only and no-stop trials (Mirabella et al., 2008, 2013). In the present study, the pattern of results found for go-only trials and no-stop trials, both on RTs and on saccadic duration, closely resembles the ones found with arm movements, even though saccades have a “point of no return” after which movement preparation is no longer controllable. Therefore, our results suggest that some compensation, possibly related to proactive adjustments, takes place even for saccadic movements.

## Conclusion

The present study shows that the inhibition of a saccade which was initiated and stopped by social cues was more resistant to suppression than a saccade initiated and stopped by non-social cues. In fact, we found that saccadic inhibitory control took longer when the task required the suppression of a saccade initiated by the gaze shift of another person and inhibited by the eyes closure rather than one instructed by a change in color of a peripheral target and inhibited by a change of color of a central picture. Thus, for the first time, we showed that overcoming a social signal requires more effort than overcoming a non-social one. We argue that this is likely due to the social and communicative intrinsic value of gaze direction.

## Conflict of Interest Statement

The authors declare that the research was conducted in the absence of any commercial or financial relationships that could be construed as a potential conflict of interest.

## References

[B1] Band G. P. van der Molen M. W. Logan G. D. (2003). Horse-race model simulations of the stop-signal procedure. Acta Psychol. (Amst.) 112, 105–142. 10.1016/s0001-6918(02)00079-3 12521663

[B2] Baron-Cohen S. (1995). Mindblindness: An Essay on Autism and Theory of Mind. Cambridge, MA: MIT Press.

[B3] Boucher L. Palmieri T. J. Logan G. D. Schall J. D. (2007). Inhibitory control in mind and brain: an interactive race model of countermanding saccades. Psychol. Rev. 114, 376–397. 10.1037/0033-295X.114.2.376 17500631

[B4] Brainard D. H. (1997). The psychophysics toolbox. Spat. Vis. 10, 433–436. 10.1163/156856897x00357 9176952

[B5] Ciardo F. Marino B. F. M. Rossetti A. Actis-Grosso R. Ricciardelli P. (2013). “Face age and social status exert different modulatory effects on gaze following behaviour,” in Proceedings of the 35th Annual Conference of the Cognitive Science Society, eds Knauff M. Pauen M. Sebanz N. Wachsmuth I. (Austin, TX: Cognitive Science Society), 2058–2062.

[B6] Ciardo F. Marino B. F. M. Actis-Grosso R. Rossetti A. Ricciardelli P. (2014). Face age modulates gaze following in young adults. Sci. Rep. 4:4746. 10.1038/srep04746 24752250 PMC3994443

[B7] Cohen J. (1988). Statistical Power Analysis for the Behavioral Sciences. (2nd Edn.) Hillsdale, NJ: Erlbaum.

[B8] Dalmaso M. Pavan G. Castelli L. Galfano G. (2012). Social status gates social attention in humans. Biol. Lett. 8, 450–452. 10.1098/rsbl.2011.0881 22090207 PMC3367721

[B9] De Jong R. Coles M. G. Logan G. D. Gratton G. (1990). In search of the point of no return: the control of response processes. J. Exp. Psychol. Hum. Percept. Perform. 16, 164–182. 10.1037/0096-1523.16.1.164 2137517

[B10] Deaner R. O. Platt M. L. (2003). Reflexive social attention in monkeys and humans. Curr. Biol. 13, 1609–1613. 10.1016/j.cub.2003.08.025 13678591

[B11] Deaner R. O. Shepherd S. V. Platt M. L. (2007). Familiarity accentuates gaze cuing in women but not men. Biol. Lett. 3, 64–67. 10.1098/rsbl.2006.0564 17443967 PMC2373814

[B12] Driver J. Davis G. Ricciardelli P. Kidd P. Maxwell E. Baron-Cohen S. (1999). Gaze perception triggers reflexive visuospatial orienting. Vis. Cogn. 6, 509–540. 10.1080/135062899394920

[B13] Farroni T. Csibra G. Simion F. Johnson M. H. (2002). Eye contact detection in humans from birth. Proc. Natl. Acad. Sci. U S A 99, 9602–9605. 10.1073/pnas.152159999 12082186 PMC123187

[B14] Farroni T. Massaccesi S. Pividori D. Johnson M. H. (2004). Gaze following in newborns. Infancy 5, 39–60. 10.1207/s15327078in0501_2

[B15] Federico P. Mirabella G. (2014). Effects of probability bias in response readiness and response inhibition on reaching movements. Exp. Brain Res. 232, 1293–1307. 10.1007/s00221-014-3846-8 24477763

[B16] Fischer B. Weber H. (1996). Effects of pro-cues on error rate and reaction times of antisaccades in human subjects. Exp. Brain Res. 109, 507–512. 10.1007/bf00229636 8817282

[B17] Friesen C. K. Ristic J. Kingstone A. (2004). Attentional effects of counterpredictive gaze and arrow cues. J. Exp. Psychol. Hum. Percept. Perform. 30, 319–329. 10.1037/0096-1523.30.2.319 15053691

[B18] Friesen C. K. Kingstone A. (1998). The eyes have it! Reflexive orienting is triggered by nonpredictive gaze. Psychonom. Bull. Rev. 5, 490–495. 10.3758/bf03208827

[B19] Friesen C. K. Kingstone A. (2003). Abrupt onsets and gaze direction cues trigger independent reflexive attentional effects. Cognition 87, B1–B10. 10.1016/s0010-0277(02)00181-636 12499107

[B20] Frischen A. Bayliss A. P. Tipper S. P. (2007). Gaze cueing of attention: visual attention, social cognition and individual differences. Psychol. Bull. 133, 694–724. 10.1037/0033-2909.133.4.694 17592962 PMC1950440

[B21] Gobel M. S. Kim H. S. Richardson D. C. (2015). The dual function of social gaze. Cognition 136, 359–364. 10.1016/j.cognition.2014.11.040 25540833

[B22] Gregory N. J. Hodgson T. L. (2012). Giving subjects the eye and showing them the finger: socio-biological cues and saccade generation in the anti-saccade task. Perception 41, 131–147. 10.1068/p7085 22670343

[B23] Hanes D. P. Carpenter R. H. (1999). Countermanding saccades in humans. Vis. Res. 39, 2777–2791. 10.1016/s0042-6989(99)00011-5 10492837

[B24] Hanes D. P. Schall J. D. (1996). Neural control of voluntary movement initiation. Science. 274, 427–430. 10.1126/science.274.5286.427 8832893

[B25] Hanes D. P. Patterson W. F. Schall J. D. (1998). Role of frontal eye fields in countermanding saccades: visual, movement and fixation activity. J. Neurophysiol. 79, 817–834. 9463444 10.1152/jn.1998.79.2.817

[B26] He P. Y. Kowler E. (1989). The role of location probability in the programming of saccades: implications for “center-of-gravity” tendencies. Vision Res. 29, 1165–1181. 10.1016/0042-6989(89)90063-1 2617863

[B27] Jonides J. (1981). “Voluntary versus automatic control over the mind’s eye’s movement,” in Attention and Performance IX, eds Long J. Baddeley A. (Hillsdale, NJ: Erlbaum), 187–203.

[B28] Koval M. J. Thomas B. Everling S. (2005). Task-dependent effects of social attention on saccadic reaction times. Exp. Brain Res. 167, 475–480. 10.1007/s00221-005-0206-8 16283398

[B29] Kuhn G. Kingstone A. (2009). Look away! Eyes and arrows engage oculomotor responses automatically. Atten. Percept. Psychophys. 71, 314–327. 10.3758/app.71.2.314 19304621

[B30] Lakens D. (2013). Calculating and reporting effect sizes to facilitate cumulative science: a practical primer for t-tests and ANOVAs. Front. Psychol. 4:863. 10.3389/fpsyg.2013.00863 24324449 PMC3840331

[B31] Langdon R. Smith P. (2005). Spatial cueing by social versus nonsocial directional signals. Vis. Cogn. 12, 1497–1527. 10.1080/13506280444000805

[B32] Levitt H. (1971). Transformed up-down methods in psychoacoustics. J. Acoust. Soc. Am. 49, 467–477. 10.1121/1.1912375 5541744

[B33] Logan G. D. (1981). “Attention, automacity and the ability to stop a speeded choice response,” in Attention and Performance IX, eds Long J. Baddeley A., (Hillsdale, NJ: Erlbaum), 205–222.

[B34] Logan G. D. (1994). “On the ability to inhibit thought and action: a users’ guide to the stop signal paradigm,” in Inhibitory Processes in Attention, Memory and Language, eds Dagenbach D. Carr T. H. (San Diego, CA: Academic), 189–239.

[B35] Logan G. D. Cowan W. B. (1984). On the ability to inhibit thought and action: a theory of an act of control. Psychol. Rev. 91, 295–327. 10.1037/0033-295x.91.3.295 24490789

[B36] Logan G. D. Cowan W. B. Davis K. A. (1984). On the ability to inhibit simple and choice reaction time responses: a model and a method. J. Exp. Psychol. Hum. Percept. Perform. 10, 276–291. 10.1037/0096-1523.10.2.276 6232345

[B37] Mansfield E. Farroni T. Johnson M. (2003). Does gaze perception facilitate overt orienting? Vis. Cogn. 10, 7–14. 10.1080/713756671

[B38] Mirabella G. (2014). Should I stay or should I go? Conceptual underpinnings of goal-directed actions. Front. Syst. Neurosci. 8:206. 10.3389/fnsys.2014.00206 25404898 PMC4217496

[B39] Mirabella G. Iaconelli S. Modugno N. Giannini G. Lena F. Cantore G. (2013). Stimulation of subthalamic nuclei restores a near normal planning strategy in Parkinson’s patients. PLos One 8:e62793. 10.1371/journal.pone.0062793 23658775 PMC3643906

[B40] Mirabella G. Pani P. Ferraina S. (2008). Context influences on the preparation and execution of reaching movements. Cogn. Neuropsychol. 25, 996–1010. 10.1080/02643290802003216 19378414

[B41] Mirabella G. Pani P. Paré M. Ferraina S. (2006). Inhibitory control of reaching movements in humans. Exp. Brain Res. 174, 240–255. 10.1007/s00221-006-0456-0 16636792

[B42] Moore C. Dunham P. (1995). Joint Attention: Its Origins and Role in Development. Hillsdale, NJ: Lawrence Erlbaum Associates Inc.

[B43] Nummenmaa L. Hietanen J. K. (2006). Gaze distractors influence saccadic curvature: evidence for the role of the oculomotor system in gaze-cued orienting. Vision Res. 46, 3674–3680. 10.1016/j.visres.2006.06.004 16901525

[B44] Ohlsen G. van Zoest W. van Vugt M. (2013). Gender and facial dominance in gaze cuing: emotional context matters in the eyes that we follow. PLoS One 8:e59471. 10.1371/journal.pone.0059471 23573199 PMC3616071

[B45] Ottes F. P. Van Gisbergen J. A. Eggermont J. J. (1985). Latency dependence of colour-based target vs. nontarget discrimination by the saccadic system. Vision Res. 25, 849–862. 10.1016/0042-6989(85)90193-2 4024483

[B46] Pelli D. G. (1997). The VideoToolbox software for visual psychophysics: transforming numbers into movies. Spat. Vis. 10, 437–442. 10.1163/156856897x00366 9176953

[B47] Posner M. I. (1980). Orienting of attention. Q. J. Exp. Psychol. 32, 3–25. 7367577 10.1080/00335558008248231

[B480] R Development Core Team. (2014). R: A Language and Environment for Statistical Computing. Vienna: R Foundation for Statistical Computing Available online at: http://www.R-project.org

[B48] Reingold E. M. Stampe D. M. (2002). Saccadic inhibition in voluntary and reflexive saccades. J. Cogn. Neurosci. 14, 371–388. 10.1162/089892902317361903 11970798

[B49] Reingold E. M. Stampe D. M. (2000). “Saccadic inhibition and gaze contingent research paradigms,” in Reading as a Perceptual Process, eds Kennedy A. Radach R. Heller D. Pynte J. (Amsterdam: Elsevier), 119–145.

[B50] Ricciardelli P. Betta E. Pruner S. Turatto M. (2009). Is there a direct link between gaze perception and joint attention behaviours? Effects of gaze contrast polarity on oculomotor behaviour. Exp. Brain Res. 194, 347–357. 10.1007/s00221-009-1706-8 19183970

[B51] Ricciardelli P. Bricolo E. Aglioti S. M. Chelazzi L. (2002). My eyes want to look where your eyes are looking: exploring the tendency to imitate another individual’s gaze. Neuroreport 13, 2259–2264. 10.1097/00001756-200212030-00018 12488807

[B52] Ricciardelli P. Carcagno S. Vallar G. Bricolo E. (2013). Is gaze following purely reflexive or goal-directed instead? Revisiting the automaticity of orienting attention by gaze cues. Exp. Brain Res. 224, 93–106. 10.1007/s00221-012-3291-5 23064809

[B53] Richardson D. C. Gobel M. S. (2015). “Social attention,” in Handbook of Attention, eds Fawcett J. Risko E. Kingstone A. (Cambridge, MA: MIT Press), 46–61.

[B54] Scangos K. W. Stuphorn V. (2010). Medial frontal cortex motivates but does not control movement initiation in the countermanding task. J. Neurosci. 30, 1968–1982. 10.1523/jneurosci.4509-09.2010 20130204 PMC4041090

[B55] Schall J. D. (2013). Production, control and visual guidance of saccadic eye movements. ISRN Neurol. 2013:752384. 10.1155/2013/752384 24260720 PMC3821953

[B56] Teufel C. Alexis D. M. Clayton N. S. Davis G. (2010). Mental-state attribution drives rapid, reflexive gaze following. Atten. Percept. Psychophys. 72, 695–705. 10.3758/app.72.3.695 20348576

[B58] Verbruggen F. Logan G. D. (2009). Proactive adjustments of response strategies in the stop-signal paradigm. J. Exp. Psychol. Hum. Percept. Perform. 35, 835–854. 10.1037/a0012726 19485695 PMC2690716

[B59] Watson A. B. Pelli D. G. (1983). Quest: a bayesian adaptive psychometric method. Percept. Psychophys. 33, 113–120. 10.3758/bf03202828 6844102

[B60] Wiese E. Wykowska A. Zwickel J. Müller H. J. (2012). I see what you mean: how attentional selection is shaped by ascribing intentions to others. PLoS One 7:e45391. 10.1371/journal.pone.0045391 23049794 PMC3458834

[B61] Wykowska A. Wiese E. Prosser A. Müller H. J. (2014). Beliefs about the minds of others influence how we process sensory information. PLoS One 9:e94339. 10.1371/journal.pone.0094339 24714419 PMC3979768

[B62] Wolohan F. D. Crawford T. J. (2012). The anti-orienting phenomenon revisited: effects of gaze cues on antisaccade performance. Exp. Brain Res. 221, 385–392. 10.1007/s00221-012-3180-y 22797785

