# Supplementary material for: Can we resist another person’s gaze?
Source: Front Behav Neurosci. 2015 Sep 30;9:258. doi: 10.3389/fnbeh.2015.00258 (PMC4623777; doi:10.3389/fnbeh.2015.00258)
Supplement: Supplementary file 1 [file Presentation_1.pdf]

Can we resist another person's gaze?

Barbara FM Marino[1]§, Giovanni Mirabella[2,3*]§, Rossana Actis-Grosso[1,4], Emanuela Bricolo[1,4], and Paola Ricciardelli[1,4]

[1]*Department of Psychology, University of Milano – Bicocca, Italy;*

[2]*Department of Physiology and Pharmacology, La Sapienza University Italy;*

[3]*Department of Neuroscience, IRCSS Neuromed, Pozzilli (IS), Italy;*

[4]*Milan Center for Neuroscience, Italy*

§ These authors contributed equally to this work

[*]Corresponding Author:

Giovanni Mirabella, PhD

Department of Physiology and Pharmacology 'V. Erspamer'

La Sapienza University

Piazzale Aldo Moro 5, 00185 Rome, Italy

Tel (+39) 06 49912312

e-mail: giovanni.mirabella@uniroma1.it

S1. Perceptual load control experiment

Participants

Sixteen undergraduate and graduate students (12 female, 4 male, mean age = 26.7 years, SD = 3.9) from the University of Milano-Bicocca took part in the experiment. Sample size was calculated *a priori* using GPower software (version 3.1, Universität Kiel, Germany) to obtain a statistical power greater than 0.90 (Cohen's effect size for F test = 0.10) at an α error probability of 0.05. All participants had normal or corrected-to-normal vision, had no history of neurological diseases, were unaware of the study's purpose, were all right handed and did not take part in the main experiment. All gave a written informed consent before testing. The study was conducted in accordance with the ethical standards laid down in the 1964 Declaration of Helsinki and fulfilled the ethical standard procedure recommended by the Italian Association of Psychology (AIP).

Materials and procedure

The experiment was carried out in dimly illuminated room. Participants sat approximately 116 cm away from a 27-inch LCD monitor (acer® HN274H; Resolution: 1920×1080 pixels; Refresh rate: 120 Hz) with their head placed on a chinrest in order to maintain a stable eye-to-screen distance. Eye height was adjusted so to keep the participant's gaze fixed on the center of the monitor throughout the experiment. The monitor was interfaced with an AMD Athlon™ Dual Core 2.00 GHz personal computer equipped with a NVIDIA® GeForce® GTX 560 Video Board. A keyboard was put centrally in front of the participant.

Five greyscale photos (4.31 × 1.48 deg) of the eye region of one of the author's (P.R.), bearing a neutral expression, were used as gaze stimuli. The photos depicted a closed gaze (with eyelids closed over each eye), a leftward gaze (with visible irises and pupils in the left-most position of the eye sockets), a rightward gaze (with irises and pupils in the right-most position of the eye sockets), and a direct gaze characterized by an unequal size of the eyes' pupils (either with the left pupil bigger than the right pupil or *vice versa*).

Participants were individually tested in an experimental session which lasted about 30 minutes. They were asked to perform a go/no-go task under three different conditions, namely the social condition (SC), the peripheral non-social condition (peripheral N-SC), and the central non-social condition (central N-SC). Conditions were presented in separate blocks and the order of presentation was counterbalanced across participants. Resting periods were allowed between blocks whenever requested.

The use of this design had two purposes. On the one hand, we aimed to assess the perceptual load of the go signals used in the main experiment (SC and peripheral N-SC) plus a third signal (central N-SC) in which saccades were cued from the centre of the screen rather than in the peripheral vision. On the other hand, we wanted to test whether different types of no-go signals (i.e. with a non-social nature, see below) could impact the inhibitory performance.

Each trial started with the presentation of the photo depicting the closed gaze, centrally displayed on a black background (Figure S.1). A red fixation cross (0.35× 0.35 deg) laid on the between-eyes point of the closed gaze. The gaze was flanked by two white target squares (0.7 × 0.7 deg), one to the left and the other to the right of the horizontally aligned fixation cross (eccentricity: 7.91 deg). After a variable delay of 200-700 ms (in order to avoid response habituation), the instruction cue was presented (either the go or no-go signal). In the SC, the go-signal consisted of a dynamic gaze, shifting randomly towards the left or the right peripheral target. The gaze shift was created by replacing the photo of the closed gaze with the photo of either the leftward or rightward gaze. The no-go signal was represented by a semi-transparent red filter being applied to the irises so as to make the shifting gaze as red-tinted (see Figure S.1, upper panel). By contrast, in the peripheral N-SC, the go-signal consisted of a change in colour of the left or right peripheral target from white to red, whereas the no-go signal consisted of a semi-transparent red filter being applied to the central photo so as to make the closed gaze as red-tinted (see Figure S.1, middle panel). It is noteworthy that the go-signals used in the present SC and peripheral N-SC were the same as those used in the no-stop trials of the main experiment. Finally, in the central N-SC, the go-signal

consisted of the opening of the eyes which was created by replacing the photo of the closed gaze with the photo of a direct gaze displaying the left or right pupil bigger than the other pupil. The no-go signal instead was represented by a semi-transparent red filter being applied to the irises so as to make the direct gaze as red-tinted (see Figure S.1, lower panel).

When a go-signal was presented, participants were required to discriminate: 1) the direction of the shifting gaze (in the SC); 2) the location (left or right) of the target that changed colour (in the peripheral N-SC); and 3) the location (left or right) of the eye with the bigger pupil (for half of the participants) or the smaller one (for the other half of participants), by pressing spatially corresponding keys (i.e. left or right) with their index fingers on the computer keyboard. By contrast, at the presentation of the no-go signal, participants were instructed to refrain from responding. All participants were asked to respond as quickly and accurately as possible. The maximum time allowed to respond was 1500 ms, thereafter the trial was aborted. The intertrial interval was 1500 ms during which the PC screen remained blank. Participants could take a break, if needed, after every 25 trials.

In each of the three experimental blocks, participants performed 96 trials (a random mix of 67% go trials and 33% no-go trials), plus 18 practice trials, for a total of 342 trials. During the practice trials, participants received visual feedback after pressing the wrong key in a go trial (i.e. the word “ERROR” appeared on the screen), after pressing a key in a no-go trial (“ERROR”), after taking more than 1500 ms to respond in a go trial (“TOO SLOW”), or after a correct response (“CORRECT”). The feedback remained visible for 1500 ms. Stimulus presentation and response time collection were controlled by E-Prime software, version 2.0. (Psychology Software Tools, Inc.).

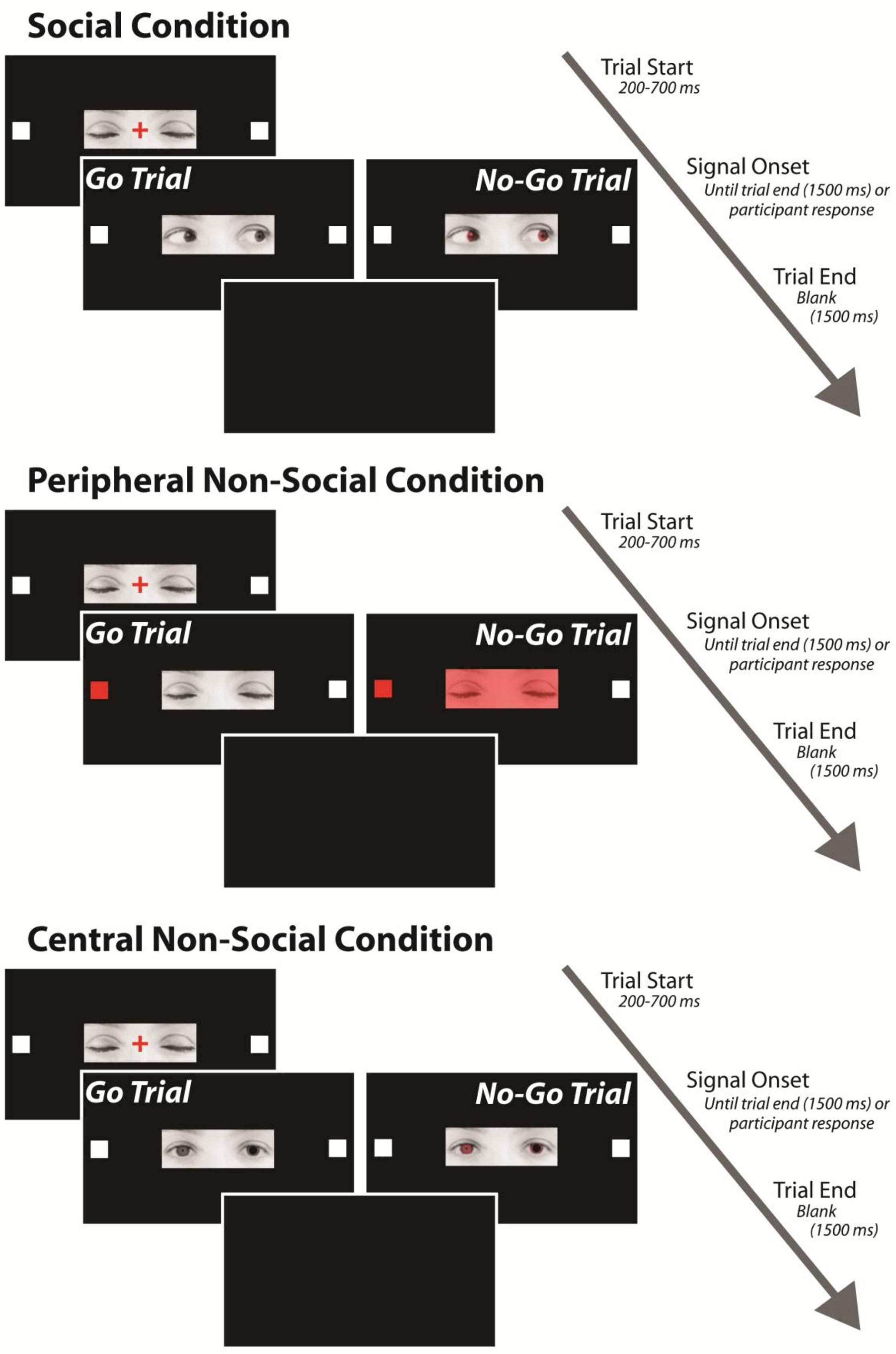

Figure S.1 – Schematic representation of the experimental procedure for social, peripheral non-social, and central non-social conditions.

Results and discussion

Trials with a wrong answer were classified errors. Errors included all cases in which participants responded to a go trial by pressing the wrong key (0.8 ± 1.5 % of go trials across all subjects) and cases in which participants responded to a no-go trial (5.4 ± 5.6 % of no-go trials across all subjects). Unfortunately, such a low percentage of errors in the no-go trials did not allow us to highlight any difference in the efficiency of the no-go signals used in inhibiting response in the present task. Further experiments are needed to disentangle this issue.

Median values of response times (RTs) measured for correct trials were calculated for each experimental condition (i.e. SC, peripheral N-SC, and central N-SC). These data were submitted to a one-way repeated measures analysis of variance (ANOVA) with condition as the within-subject variable. The ANOVA revealed that the effect of condition [$F(2,30) = 38.14$, $p < .001$) was significant (Figure S.2). The post-hoc tests (pairwise comparisons with Bonferroni correction) showed that RTs in central N-SC were significantly longer than those in both peripheral N-SC ($p < .0001$) and SC ($p < .0001$), which did not differ from each other ($p = .76$). This result clearly indicates that the go signals used in the SC and N-SC of the main experiment (respectively the SC and peripheral N-SC in the present control experiment) presented a similar perceptual load. In other words, when the response needs to be withheld as is the case in the go/no-go task, detecting the direction of the gaze required a similar amount of perceptual processing than detecting a change in the target colour. Therefore, the lengthening of the stop signal reaction time (SSRT) found in the main experiment for SC relative to N-SC is unlikely to be ascribed to the increased perceptual load needed to process the two go signals: the gaze shift and the lighting up of the peripheral target. Crucially, the present results support further our choice of employing a typical exogenous cue (a change in colour of a peripheral cue) as control for our social cue (a gaze shift) in the main experiment. The speed of the correct answers made in response to both cues, in fact, is similar, although that to the central biologically irrelevant cue used in the present control experiment (i.e., central N-SC) was significantly slower.

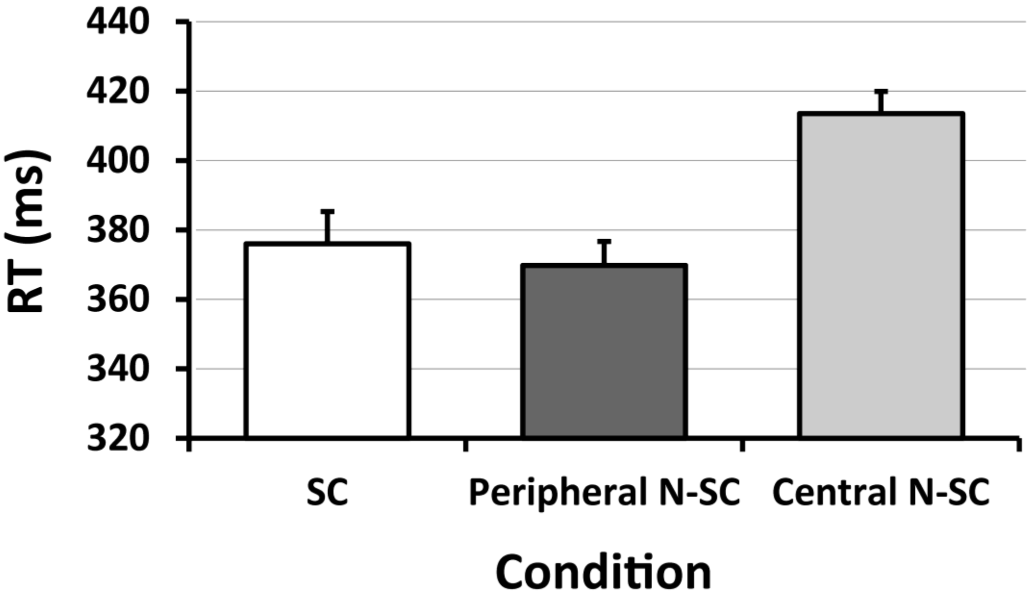

Figure S.2 – Mean reaction time measured for social (white bar), peripheral non-social (dark grey bar), and central non-social (light grey bar) conditions. The error bars represent the standard error.
